# Intratumoral Heterogeneity and Immune Response Indicators to Predict Overall Survival in a Retrospective Study of HER2-Borderline (IHC 2+) Breast Cancer Patients

**DOI:** 10.3389/fonc.2021.774088

**Published:** 2021-11-11

**Authors:** Gedmante Radziuviene, Allan Rasmusson, Renaldas Augulis, Ruta Barbora Grineviciute, Dovile Zilenaite, Aida Laurinaviciene, Valerijus Ostapenko, Arvydas Laurinavicius

**Affiliations:** ^1^ National Center of Pathology, Affiliate of Vilnius University Hospital Santaros Clinics, Vilnius, Lithuania; ^2^ Institute of Biosciences, Life Sciences Center, Vilnius University, Vilnius, Lithuania; ^3^ Faculty of Medicine, Institute of Biomedical Sciences, Vilnius University, Vilnius, Lithuania; ^4^ Department of Breast Surgery and Oncology, National Cancer Institute, Vilnius, Lithuania

**Keywords:** HER2, breast cancer, intratumoral heterogeneity, CD8, immune response, tumor microenvironment, digital pathology

## Abstract

Breast cancer (BC) categorized as human epidermal growth factor receptor 2 (HER2) borderline [2+ by immunohistochemistry (IHC 2+)] presents challenges for the testing, frequently obscured by intratumoral heterogeneity (ITH). This leads to difficulties in therapy decisions. We aimed to establish prognostic models of overall survival (OS) of these patients, which take into account spatial aspects of ITH and tumor microenvironment by using hexagonal tiling analytics of digital image analysis (DIA). In particular, we assessed the prognostic value of Immunogradient indicators at the tumor–stroma interface zone (IZ) as a feature of antitumor immune response. Surgical excision samples stained for estrogen receptor (ER), progesterone receptor (PR), Ki67, HER2, and CD8 from 275 patients with HER2 IHC 2+ invasive ductal BC were used in the study. DIA outputs were subsampled by HexT for ITH quantification and tumor microenvironment extraction for Immunogradient indicators. Multiple Cox regression revealed HER2 membrane completeness (HER2 MC) (*HR*: 0.18, *p* = 0.0007), its spatial entropy (*HR*: 0.37, *p* = 0.0341), and ER contrast (*HR*: 0.21, *p* = 0.0449) as independent predictors of better OS, with worse OS predicted by pT status (*HR*: 6.04, *p* = 0.0014) in the *HER2* non-amplified patients. In the *HER2*-amplified patients, HER2 MC contrast (*HR*: 0.35, *p* = 0.0367) and CEP17 copy number (*HR*: 0.19, *p* = 0.0035) were independent predictors of better OS along with worse OS predicted by pN status (*HR*: 4.75, *p* = 0.0018). In the non-amplified tumors, three Immunogradient indicators provided the independent prognostic value: CD8 density in the tumor aspect of the IZ and CD8 center of mass were associated with better OS (*HR*: 0.23, *p* = 0.0079 and 0.14, *p* = 0.0014, respectively), and CD8 density variance along the tumor edge predicted worse OS (*HR*: 9.45, *p* = 0.0002). Combining these three computational indicators of the CD8 cell spatial distribution within the tumor microenvironment augmented prognostic stratification of the patients. In the *HER2*-amplified group, CD8 cell density in the tumor aspect of the IZ was the only independent immune response feature to predict better OS (*HR*: 0.22, *p* = 0.0047). In conclusion, we present novel prognostic models, based on computational ITH and Immunogradient indicators of the IHC biomarkers, in HER2 IHC 2+ BC patients.

## Introduction

Breast cancer (BC) is a complex and diverse disease with distinct clinical, pathological, and molecular characteristics. The multifaceted nature of the disease leads to diverse clinical outcomes and therapeutic responses. BC has been classified into several biologically distinct subtypes: luminal A, luminal B, human epidermal growth factor receptor 2 (HER2)-enriched (HER2), basal-like, and normal-like by gene expression profiling analysis (1, 2), requiring different treatment strategies. This categorization of the BC subtypes has been adapted for clinical practice and is mainly based on immunohistochemistry (IHC) assessment of estrogen receptor (ER), progesterone receptor (PR), HER2, and Ki67 expression.

Routinely used predictive features, including clinicopathological parameters (age, tumor size, lymph node status, and histological grade) and biomarkers (ER, PR, and HER2) are insufficient for personalized clinical decisions in BC patients (3). Novel prognostic BC biomarkers have been intensively investigated as recently reviewed by Wu et al. (4). In particular, robust biomarkers are in demand for HER2-positive disease to improve selection of patients for current and emerging therapies of HER2-positive metastatic BC (5) as well as for prediction of resistance for anti-HER2 therapies, recurrence (6, 7), and particular consequences of the disease (8). Novel approaches based on pathology image analytics and machine learning methods open new perspectives for predictive modeling and clinical decision support (9, 10). Importantly, both molecular and image-based biomarkers can be explored and validated using The Cancer Genome Atlas (TCGA) Data Portal (11).

HER2 amplification and overexpression occur in approximately 15%–20% of invasive BC cases and are associated with worse patient survival as compared with non-amplified *HER2* BC (12–15). A positive HER2 status predicts better effect of HER2-targeted therapies, and therefore, its accurate detection is essential for treatment decisions (16, 17).

While the majority of tumors can be categorized as either HER2-positive or HER2-negative by IHC and *in situ* hybridization (ISH) techniques, which are regarded as the standard methods to assess HER2 status in BC, borderline tumors do account for up to 18% of BCs (18, 19) and present challenges for patient assessment and therapy choices. In 2018, the American Society of Clinical Oncology (ASCO) and College of American Pathologists (CAP) updated the guidelines for HER2 testing with revised criteria for HER2 IHC borderline (IHC 2+) classification. This mainly focused on less common fluorescence ISH (FISH) patterns (ASCO/CAP groups 2, 3, and 4) and recommended to integrate them with a concomitant IHC review for a final HER2 result determination (20). The ambiguous FISH equivocal group (18), which poses therapeutic dilemmas, was removed, which resulted in an increased frequency of HER2-negative cases (21–24). Nevertheless, some studies report that HER2 equivocal tumors present similar clinical behaviors to the HER2-negative BC (25, 26), while others find differences in clinicopathological and prognostic aspects between these two categories (23). This suggests that the equivocal category represents an intermediate state between HER2-positive and HER2-negative tumors (27, 28).

Approximately 15%–30% of IHC 2+ cases are *HER2*-amplified (29), while the remaining IHC 2+ and IHC 1+ *HER2* non-amplified tumors were recently designated as a relatively common “HER2-low” category, accounting for approximately 40%–55% of BC (30–32). This concept becomes important with the advent of a new generation of anti-HER2 agents. Specifically, ongoing clinical trials have demonstrated high efficacy of antibody–drug conjugates that are designed to target and deliver chemotherapy inside cancer cells in this particular subset of BC patients (33–35). The HER2-low BC group is not formally defined at present, but if treatment options will become available, the current dichotomous HER2 guidelines will have to be revised further to distinguish truly HER2-negative from HER2-low breast cancer (31).

The intratumoral heterogeneity (ITH) of HER2, at both protein expression and gene amplification levels, is a common feature of HER2-borderline tumors, which further complicates the assessment of HER2 status (36–40). In addition to the heterogeneous HER2 expression, the variable expression of hormone receptors (HRs) also contributes to the ITH and may further affect clinical outcomes and responses to treatment of BC (41, 42). Potential interactions between HR and HER2 signaling pathways, which could impact development of resistance to endocrine and anti-HER2 therapies, have been highlighted by several preclinical and clinical studies (43–48).

Current pathological IHC methods are based on the assessment of a proportion of HER2-positive tumor cells; however, ITH of HER2 expression may present a challenge in some tumors to be categorized with a single value (0, 1+, 2+, and 3+). Digital image analysis (DIA) has opened new opportunities in HER2 IHC assessment by providing biomarker quantification with increased accuracy, precision, reproducibility, and capacity (49–55). Studies have demonstrated that DIA can reliably distinguish HER2 IHC negative (0–1+) and positive (3+) cases and reduce the proportion of IHC 2+ cases (51–54). Importantly, continuous data and spatial aspects of IHC biomarker distribution can be revealed by DIA (56–58). Several diversity metrics (the Shannon entropy (59), the Simpson index (60), and Rao’s quadratic entropy (61) have been adapted for molecular, genetic, and microenvironmental heterogeneity assessments in BC) (62–65). Potts et al. examined HER2 expression ITH in BC by combining semiquantitative analysis with ecology diversity statistics (64). Both cell-level and tumor-level heterogeneities were evaluated, but the authors had doubts about the insufficient number of regions sampled to make an assessment of heterogeneity at a tumor level. Several recent studies (56–58) showed a successful assessment of ITH of IHC biomarkers in whole slide images (WSIs) based on hexagonal grid subsampling of DIA data; importantly, this methodology enabled retrieval of prognostically informative spatial heterogeneity indicators of tissue biomarker expression.

Although ITH may challenge the efficacy of therapy, it may be also associated with favorable prognostic effects, since a greater mutational load could lead to an increased tumor neo-antigen generation that attracts immune cells and stimulate antitumor immunity (66, 67). However, immunogenicity is different among BC subtypes, with generally higher mutational load, higher numbers of tumor-infiltrating lymphocytes (TILs), and higher programmed death-ligand 1 (PD-L1) expression in triple-negative and lower in HR-positive subtypes (68–71). These differences may impact the efficacy of therapy with immune checkpoint inhibitors with significant responses achieved only in patients with triple-negative BC so far (72–74).

TILs have been recognized as a potential biomarker of survival on BC patients (75, 76); however, their prognostic significance varies in BC types (70). A positive prognostic role of CD8 cells has been demonstrated in ER-negative and triple-negative BC (77–79), but its prognostic value in HR-positive BC remains unclear (70, 80). Recent studies have shown that the distance between immune cells and cancer cells is clinically and prognostically important in BC (81–84). The methods for assessing TILs and their spatial distributions have been the focus of many studies. Recently, Krijgsman et al. (85) first applied an automated deep learning approach that identifies the tumor boundary and detects CD8-positive cells in IHC images, and then they analyzed the spatial distribution of CD8 lymphocytes in ER-positive invasive BC. They found that only the SD of the CD8 density (but not the mean of CD8 density) distribution was significantly associated with better survival, hypothesizing that it reflects the contribution from local high-density areas. In another study (84), the immune scores of cell abundance and spatial heterogeneity were quantified using a combination of fully automated H&E-stained image analysis and spatial statistics. High immune spatial scores, but not the abundance scores, were associated with poor prognosis in ER positive BC. Rasmusson et al. proposed a hexagonal grid-based methodology to automatically detect the tumor–host interface zone (IZ) and compute the immune cell density profile across the interface. The computed Immunogradient indicators provided the independent prognostic value in HR-positive breast and colorectal cancer patients (86).

In our study, we investigated ITH and immune response properties of HER2 IHC 2+ borderline BC patients with regard to their prognostic value. We utilized image DIA of IHC for ER, PR, Ki67, HER2, and CD8, with subsequent hexagonal grid analytics to extract combined prognostic overall survival (OS) models in HER2 IHC 2+ FISH-negative and FISH-positive patients.

## Materials and Methods

### Patients and Samples

This retrospective study included 275 patients, selected from an initial set of 302 patients with invasive ductal breast carcinoma diagnosed as HER2 borderline by IHC (IHC 2+), treated at the National Cancer Institute of Lithuania and investigated at the National Center of Pathology, affiliate of the Vilnius University Hospital Santaros Klinikos, between September 2012 and March 2017. The selected patients met the following criteria: 1) patients diagnosed with invasive ductal carcinoma; and 2) HER2 IHC 2+ cases assessed by a pathologist, tested routinely by HER2 FISH and ER, PR, Ki67, and CD8 IHC slides available for DIA. The cases without paraffin blocks available for CD8 IHC staining and available follow-up data were excluded (15 and 12 cases, respectively). Clinical and pathology information was collected retrospectively from the medical records. The study was approved by the Lithuanian Bioethics Committee (reference number: 40, April 26, 2007, updated on March 18, 2013, and on July 4, 2016).

### Immunohistochemistry

Formalin-fixed paraffin-embedded (FFPE) surgical excision samples tissue were cut at 3µm thickness and mounted on positively charged slides and for IHC staining by Roche Ventana BenchMark ULTRA automated slide stainer (Ventana Medical Systems, Tucson, AZ). IHC for ER, PR, and HER2 was performed using ready-to-use antibodies (SP1, 1E2, and 4B5, respectively, Ventana (Tucson, Arizona, USA); for Ki67 and CD8—MIB-1, Dako (Glostrup, Denmark; dilution 1:100) and C8/144B, Dako (Glostrup, Denmark; dilution 1:100) antibodies, respectively. Visualization of ER, PR, Ki67, HER2, and CD8 was performed with the ultraView Universal DAB Detection kit (Ventana Medical Systems, Tucson, Arizona, USA). Tissue sections were counterstained with Mayer’s hematoxylin.

HER2 expression was scored as 0 (no staining, or incomplete membrane staining that is faint or barely perceptible and within ≤10% of the invasive tumor cells); 1+ (incomplete membrane staining that is faint or barely perceptible and within >10% of the invasive tumor cells); 2+ (weak-to-moderate complete membrane staining observed in >10% of tumor cells); or 3+ (circumferential membrane staining that is complete, is intense, and in >10% of tumor cells) according to the 2018 ASCO/CAP guidelines (20). IHC 0 and IHC 1+ were defined as HER2 negative, IHC 2+ was categorized as HER2 borderline, and IHC 3+ was categorized as HER2 positive.

### Fluorescence *In Situ* Hybridization

*HER2* FISH was performed on FFPE sections using the PathVysion *HER2* DNA probe kit and Paraffin pretreatment kit (Abbott-Vysis, Inc., Downers Grove, IL, USA) as described in detail previously (87). Briefly, 4 µm thick sections were mounted on positively charged slides and dried overnight at 56°C. Subsequently, deparaffinization, dehydration, and pretreatment procedures were performed. After the digestion with protease, the hybridization mixture containing two fluorescently labeled DNA probes recognizing the *HER2* locus (17q11.2-q12) and the centromeric region of CEP17 (17p11.1-q11.1) was applied to the target tissue. Denaturation and hybridization were performed in a hybridizer (Dako Diagnostics, Glostrup, Denmark). Then slides were washed, counterstained with DAPI, and coverslipped (Invitrogen Corporation, Carlsbad, USA). The samples were analyzed using a fluorescence microscope (Zeiss, Axio Imager.Z2, Gottingen, Germany) equipped with single-pass filters for DAPI, HER2, and CEP17, under a 63× oil immersion objective. All tumors were tested routinely by dual-probe FISH assay for final HER2 classification according to the ASCO/CAP guidelines (20).

### Digital Image Acquisition, Analysis, and Calculation of Indicators

For the analysis of ER, PR, Ki67, and HER2, sections were scanned using a ScanScope XT Slide Scanner (Leica Aperio Technologies, Vista, CA, USA) at ×20 objective magnification (0.5 μm per pixel); CD8 IHC slides were scanned using an Aperio AT2 Slide Scanner (Leica Biosystems, Buffalo Grove, IL, USA) at ×20 objective magnification (0.5 μm per pixel). The DIA was performed on the WSIs with HALO™ software (version 3.0311.174; Indica Labs, Corrales, NM, USA) by three operators (RG, RA, and GR). Initially, the tissue was classified into the tumor, stroma, and background (consisting of glass, necrosis, and artifacts) by HALO AI™ classifier. Subsequently, the HALO Multiplex IHC and Membrane algorithms (versions 1.2 and 1.4, respectively) were applied to obtain coordinates of the cells in the IHC WSI. For quality assurance, all image analysis results were approved by the breast pathologist (RG).

Positive cell percentages for ER, PR, and Ki67 and the percentages of HER2 2+ and 3+ cells along with the cell membrane completeness (MC) indicator were obtained by the HALO DIA. ITH indicators were computed by systematic subsampling of the HALO DIA data using hexagonal tiling arrays as described previously in (56). Briefly, the cells were assigned to 825-pixel-sized hexagons (hexagon side length 257 µm) according to their extracted coordinates. Hexagons containing fewer than 50 cells were regarded as insufficient sampling and were excluded from further analyses. Subsequently, the percentages of ER, PR, Ki67, and HER2-positive cells were calculated for each hexagon to be ranked into 10 intervals (0%–10%, >10%–20%, >20%–30%, >30%–40%, >40%–50%, >50%–60%, >60%–70%, >70%–80%, >80%–90%, and >90%–100%). Based on the ranks, a co-occurrence matrix was constructed to compute Haralick’s texture indicators (contrast, dissimilarity, entropy, energy, and homogeneity) (88). The intratumoral distributions of ER, PR, Ki67, and HER2 expression were assessed for bimodality by Ashman’s D indicator as described previously (57).

The automated extraction of the IZ and Immunogradient indicators is described in detail in (86). In our study, an IZ width of seven hexagon ranks (hexagon side length 65 μm) was used. CD8 cell density was calculated in both 1) the WSI stroma and tumor areas and 2) within the tumor–stroma IZ, which consists of stroma (S), tumor (T), and tumor edge (TE) aspects. Subsequently, Immunogradient indicators (center of mass (CM) and immunodrop) representing CD8 cell density profiles across the IZ were computed. The CM indicator reflects CD8 cell density increase towards the tumor within the IZ, while the immunodrop indicator reflects an abrupt decrease of CD8 cell density across the TE (IZ rank 0) from stroma (IZ rank −1) to tumor (IZ rank 1), represented by the CD8 cell density ratio between rank −1 and rank 1.

### Statistical Analysis

All continuous variables were tested for normal distribution by Kolmogorov–Smirnov test and compared by two-tiled Student’s *t*-test (for normally distributed variables) or the Mann–Whitney *U* test (for non-normally distributed variables). A log-transformation was applied to normalize the asymmetric distributions of immune response variables and to meet the assumptions of parametric statistical tests; they were used in one-way ANOVA followed by Bonferroni’s post-hoc test for pairwise comparisons and a two-sided Welch’s *t*-test for homogeneity of variances. Fisher’s exact test was used to assess the differences in clinicopathological variables among the analyzed groups.

A factor analysis was performed using the factoring method based on principal component analysis; factors were retained based on the threshold of an eigenvalue of 1; lastly, a general orthogonal varimax rotation of the initial factors was applied.

The optimal cutoff value for each indicator was determined using Cutoff Finder (89) to test the predictions of OS. The Kaplan–Meier method was applied to estimate the OS distributions with the log-rank test to compare survival differences between the stratified groups. To assess the prognostic factors, univariate and multivariate analyses were performed using the Cox proportional-hazards models. The “best” subset of variables to be included in the multivariate Cox proportional-hazards models was identified by leave-one-out cross-validation (90). All *p*-values were considered significant at the <0.05 level. Statistical analyses were performed with SAS software (version 9.4; SAS Institute Inc., Cary, NC, USA); plots were generated by R (version 4.1.0).

## Results

### Clinicopathological and Follow-Up Characteristics

Clinicopathological and follow-up characteristics of the *HER2* non-amplified and *HER2*-amplified groups are summarized in **Table 1**. The median follow-up period was 64 (range 2–102) and 52 months (range 0.7–100) in the non-amplified and amplified HER2 cohorts, respectively. Forty-two patients died during the follow-up, including 22 (13.7%) and 20 (17.1%) in the non-amplified and amplified tumor subsets, respectively.

**Table 1:** Patient and tumor characteristics according to HER2 status.

Characteristic	Total (n = 275)	*HER2* non-amplified, n (%)	*HER2*-amplified status, n (%)	*p*-Value^*^
Number of patients	275	158 (57.5)	117 (42.5)	
Median age, years (range)	60 (29–92)	59 (33–86)	63 (29–92)	0.2247
Median follow up, months (range)	58 (0.7–102)	64 (2–102)	52 (0.7–100)	
Deceased	42	22 (13.7)	20 (17.1)	
Histological grade (G), n (%)				
1	22	18 (11.4)	4 (3.4)	<0.0001*
2	153	99 (62.7)	54 (46.2)	
3	100	41 (25.5)	59 (50.4)	
Tumor invasion (pT), n (%)				
T1	129	77 (48.7)	52 (44.4)	0.7578
T2	129	73 (46.2)	56 (47.9)	
T3	9	4 (2.5)	5 (4.3)	
T4	8	4 (2.5)	4 (3.4)	
Lymph node metastasis (pN), n (%)				
N0	165	96 (60.8)	69 (59)	0.3225
N1	66	41 (26)	25 (21.4)	
N2	30	16 (10.1)	14 (12)	
N3	14	5 (3.2)	9 (7.7)	

HER2, human epidermal growth factor receptor 2.

*p-Value < 0.05 is considered significant.

Of the 275 IHC 2+ patients, 158 (57.5%) were diagnosed as *HER2* non-amplified (*HER2/*CEP17 ratio <2.0; average *HER2* copy number <4.0 signals per cell), while 117 (42.5%) were *HER2*-amplified (*HER2/*CEP17 ratio ≥2.0; average *HER2* copy number ≥4.0 signals per cell) on the basis of the FISH results categorized according to 2018 ASCO/CAP guidelines (20). Fifty-nine (37.3%) FISH equivocal cases under the 2013 guidelines (18) were reclassified into *HER2* non-amplified according to the 2018 guidelines (20).

The *HER2*-amplified group revealed significantly higher histological grade (*p* < 0.001) and higher frequency of increased CEP17 copy number (*p* = 0.0002) as compared with the *HER2* non-amplified group (**Table 1**). Of note, 55 (34.8%) and 67 (57.3%) cases with CEP17 copy number ≥3 were detected in the *HER2* non-amplified and *HER2*-amplified groups, respectively. No significant differences between the groups regarding the patient age, tumor stage, and node involvement were found.

### Summary Statistics of Explored Indicators

Summary statistics of the variables in the *HER2* non-amplified and *HER2*-amplified groups are presented in **Supplementary Table 1**; the variance plots of the significant differences are presented in **Supplementary Figure 1**.

In general, expression rates of ER and PR were higher, while Ki67 was lower in the *HER2* non-amplified group. No significant difference in CD8 cell density distribution between tumor and stroma areas was observed in both the *HER2* non-amplified (*t* = 1.72, *p* = 0.0867) and *HER2*-amplified (*t* = 1.07, *p* = 0.2841) groups. Also, the mean of CD8 density within the IZ was significantly higher in the S aspect than in the T aspect in both the *HER2* non-amplified (*t* = 6.56, *p* < 0.001) and *HER2*-amplified (*t* = 6.17, *p* < 0.001) groups. The variance of CD8 cells was the highest in the S aspect, less in the TE aspect, and lowest in the T aspect of the IZ in both the *HER2* non-amplified and *HER2*-amplified groups (*p* < 0.0001) (data not shown). No significant differences of CD8 cell densities neither in tumor nor stroma areas nor inside the IZ (T, TE, and S aspects) were found between the groups. ITH (higher contrast, dissimilarity, and entropy but lower energy and homogeneity) was higher only for Ki67 in the *HER2*-amplified group.

### Factor Analysis of Immunohistochemistry, Fluorescence *In Situ* Hybridization, Immune Response, and Intratumoral Heterogeneity Indicators in *HER2* Non-Amplified and *HER2*-Amplified Groups

A factor analysis was performed on the combined set of DIA IHC, FISH, immune response, and ITH data and six orthogonally independent factors in each patient group were extracted. The patterns of the factors are plotted in **Supplementary Figures 2**, **3**, factor loadings obtained after varimax rotation are presented in **Supplementary Tables 2**, **3** for the *HER2* non-amplified and *HER2*-amplified groups, respectively.

In the *HER2* non-amplified BC cases, Factor 1 was characterized by positive loadings of the variables indicative of CD8 density within the IZ T, TE, and S aspects and was named CD8 density factor. Factor 2 showed positive loadings of HER2 FISH variables (*HER2* copy number, *HER2/*CEP17 ratio, percentage of amplified cells calculated from *HER2*/CEP17 ratio, and percentage of amplified cells calculated by *HER2* signals only) and was named the *HER2* amplification factor. Factor 3 was characterized by increasing CD8 densities towards the T aspect of the IZ (strong positive loadings of the CD8 CM and its SD) and by moderate loading of CD8 density in the T aspect; therefore, it was named the CD8 density gradient. Factor 4 was represented by the Ki67% and Ki67 entropy indicators. Factor 5 was characterized by positive loadings of two Haralick’s texture indicators, namely, HER2 MC entropy and ER contrast, along with negative loading of ER%. This factor was interpreted as HER2&ER heterogeneity factor. Factor 6 was represented by PR% and PR entropy indicators.

Similarly, in *HER2-*amplified tumors, Factor 1 was the *HER2* amplification factor, Factor 2 was the CD8 density factor, and Factor 3 (CD8 density gradient factor) was the main sources of variance. Factor 4 was characterized by strong positive loadings of Ki67% and Ki67 entropy indicators and by moderate negative loading of ER entropy. Factor 5 was represented by the percentage of both HRs along with the PR entropy. Factor 6 was characterized by strong positive loading of a single HER2 MC variable.

### Prognostic Significance of Clinicopathological Parameters, Immunohistochemistry, Fluorescence *In Situ* Hybridization, Immune Response, and Intratumoral Heterogeneity Indicators in *HER2* Non-Amplified and *HER2*-Amplified Patients

We explored the potential of the clinicopathological parameters, IHC, FISH, immune response, and ITH indicators for predicting OS of the patients by univariate survival analysis. Statistically significant indicators and their hazard ratios are presented in **Table 2**. For the *HER2* non-amplified group, higher T stage, lymph node status (pN), CD8 density in the S aspect, SD of CD8 density in the S and TE aspects, immunodrop of CD8 density, and Haralick’s texture indicators reflecting homogeneity of HER2 and HER2 MC (energy, homogeneity) were associated with shorter OS. Meanwhile, higher HER2 expression, CD8 densities in the tumor area and T aspect within IZ along with its variance, CM for CD8 density and its variance, Haralick’s texture indicators reflecting heterogeneity of HER2 and HER2 MC (contrast, dissimilarity, and entropy), and ER contrast were associated with longer OS. In the *HER2*-amplified patients, worse OS was associated with higher T stage, pN, immunodrop of CD8 density, HER2 MC homogeneity, Ki67 entropy, and PR AshD (bimodality), while in the presence of higher CEP17 copy number, the remaining Immunogradient indicators, HER2 entropy, HER2 MC contrast, and dissimilarity were associated with better OS.

**Table 2:** Univariate analysis of the impact of clinicopathological parameters, Immunogradient, IHC, FISH, and intratumoral heterogeneity indicators in *HER2* non-amplified and *HER2*-amplified patient groups on overall survival using the log-rank test.

*HER2* non-amplified group				*HER2*-amplified group			
Variables and indicators	HR	95% CI	*p*-Value	Variables and indicators	HR	95% CI	*p*-Value
pT stage (pT1–2 vs. pT3–4)	4.41	1.30–14.97	0.0173	pT stage (pT1–2 vs. pT3–4)	3.49	1.01–12.05	0.049
pN stage (pN0 vs. pN1–3)	3.2	1.30–7.86	0.0111	pN stage (pN0 vs. pN1–3)	3.2	1.31–7.83	0.011
HER2%	0.26	0.11–0.62	0.001	CEP17 copy number	0.25	0.09–0.68	0.003
HER2_MC	0.12	0.05–0.32	<0.0001	CD8_T	0.38	0.16–0.91	0.024
CD8_T	0.37	0.16–0.87	0.017	CD8_CM	0.41	0.17–0.99	0.041
CD8_CM	0.2	0.08–0.49	<0.0001	CD8_d_TE	0.37	0.15–0.89	0.021
CD8_CM_sd	0.36	0.15–0.84	0.013	CD8_d_T	0.34	0.13–0.89	0.021
CD8_d_S	3.22	0.94–11.05	0.049	CD8_d_T_sd	0.35	0.14–0.89	0.022
CD8_d_S_sd	2.65	1.08–6.51	0.027	CD8_ID	3.05	1.24–7.48	0.01
CD8_d_TE_sd	2.81	1.21–6.54	0.012	HER2_entropy	0.4	0.16–1.02	0.047
CD8_d_T	0.3	0.13–0.71	0.003	HER2_MC_contrast	0.32	0.12–0.85	0.016
CD8_d_T_sd	0.35	0.14–0.85	0.016	HER2_MC_dissimilarity	0.35	0.14–0.88	0.019
CD8_ID	3.49	1.51–8.06	0.002	HER2_MC_homogeneity	2.49	0.99–6.27	0.044
HER2_contrast	0.22	0.09–0.52	0.0002	Ki67_entropy	2.39	0.99–5.77	0.044
HER2_dissimilarity	0.21	0.08–0.55	0.0005	PR_AshD	3.72	1.35–10.26	0.006
HER2_entropy	0.23	0.10–0.56	0.0004				
HER2_energy	4.28	1.81–10.08	0.0003				
HER2_homogeneity	2.95	1.26–6.90	0.009				
HER2_MC_contrast	0.37	0.14–0.94	0.029				
HER2_MC_dissimilarity	0.36	0.14–0.92	0.025				
HER2_MC_entropy	0.31	0.13–0.72	0.004				
HER2_MC_energy	3.25	1.36–7.79	0.005				
HER2_MC_homogeneity	2.9	1.18–7.13	0.015				
ER_contrast	0.21	0.05–0.91	0.021				

AshD, Ashman’s D; CEP17, centromere enumeration probe for chromosome 17; CM, center of mass; CM_sd, SD for center of mass; ID, immunodrop; d_S, density in the stroma aspect of interface zone (IZ); d_S_sd, SD in the stroma aspect of IZ; d_T, density in the tumor aspect of IZ; d_T_sd, SD in the tumor aspect of IZ; d_TE, density in the tumor edge aspect of IZ; d_TE_sd, SD in the tumor edge aspect of IZ; HR, hazard ratio; MC, membrane completeness; T, tumor area; IHC, immunohistochemistry; FISH, fluorescence in situ hybridization.

All the variables significantly associated with outcome at a univariate analysis (*p* < 0.05, **Table 2**) were assessed for their independent prognostic value in the multivariate Cox regression models.

To investigate any added prognostic value of the indicators, three models in each group were generated from different variable sets (**Table 3**). Models 1 and 4 were obtained from the pathology and IHC data, including the ITH indicators; FISH indicators were additionally used in the *HER2*-amplified group. In models 2 and 5, the IHC CD8 density and Immunogradient indicators were added to the variables tested in models 1 and 4. Models 3 and 6 were obtained from the pathological and CD8 indicators, without inclusion of any ER, PR, Ki67, and HER2 variables.

**Table 3:** Multivariate analysis of prognostic factors associated with OS in *HER2* non-amplified (models 1, 2, and 3) and *HER2*-amplified (models 4, 5, and 6) patient groups.

*HER2* non-amplified group				*HER2*-amplified group			
	HR	95% CI	*p-*Value		HR	95% CI	*p-*Value
**Model 1 (LR: 27.1, *p* < 0.0001)**				**Model 4 (LR: 17.64, *p* = 0.0005)**			
pT stage (pT1–2 vs. pT3–4)	6.04	2.31–33.04	0.0014	pN stage (pN0 vs. pN1–3)	4.75	1.77–12.62	0.0018
HER2 MC	0.18	0.07–0.48	0.0007	HER2_MC_contrast	0.35	0.13–0.94	0.0367
HER2 MC entropy	0.37	0.15–0.93	0.0341	CEP17 copy number	0.191	0.06–0.58	0.0035
ER contrast	0.21	0.05-0.97	0.0449				
**Model 2 (LR: 56.05, *p* < 0.0001)**				**Model 5 (LR: 29.03, *p* < 0.0001)**			
pT stage (pT1–2 vs. pT3–4)	13.65	3.05–61.03	0.0006	pN stage (pN0 vs. pN1-3)	7.985	2.7–23.63	0.0002
HER2 MC	0.17	0.05–0.66	0.0102	HER2_MC_contrast	0.243	0.09–0.69	0.0077
HER2 MC entropy	0.33	0.13–0.88	0.0263	CEP17 copy number	0.135	0.04–0.44	0.0008
ER contrast	0.16	0.03–0.80	0.0258	CD8_d_T	0.117	0.04–0.37	0.0002
CD8_CM	0.223	0.08–0.64	0.0053				
CD8_d_T	0.147	0.05–0.47	0.0013				
CD8_d_TE_sd	7.82	2.63–23.28	0.0002				
**Model 3 (LR: 28.26, *p* < 0.0001)**				**Model 6 (LR: 12.52, *p* = 0.0019)**			
CD8_CM	0.14	0.04–0.47	0.0014	pN stage (pN0 vs. pN1–3)	4.55	1.72–12.06	0.0023
CD8_d_T	0.23	0.08–0.68	0.0079	CD8_d_T	0.22	0.08–0.63	0.0047
CD8_d_TE_sd	9.45	2.9–30.78	0.0002				

CEP17, centromere enumeration probe for chromosome 17; CM, center of mass; d_T, density in the tumor aspect of interface zone (IZ); d_TE_sd, SD in the tumor edge aspect of IZ; HR, hazard ratio; LR, likelihood ratio; MC, membrane completeness; OS, overall survival.

In the *HER2* non-amplified group, higher values of HER2 MC, HER2 MC entropy, and ER contrast indicators were independent features of better OS, while higher tumor stage was associated with worse OS (model 1). Model 2 revealed a marked increase of prognostic power contributed by the immune response indicators in the data set (model likelihood ratio 56.1 achieved in model 2 compared with that of 27.1 in model 1); better OS was associated with higher CD8_CM and CD8_d_T cell densities, and worse OS with higher CD8_d_TE_sd. Remarkably, models 2 and 3 included the same three immune response indicators as independent prognostic factors, reflecting different properties of the local CD8 densities within tumor microenvironment: CD8_d_T (absolute density in the tumor aspect of IZ) and CD8_CM (positive IZ density gradient towards the tumor) were both associated with longer OS, while CD8_d_TE_sd (variance of the CD8 cell density along the IZ) was a feature of worse prognosis.

For the *HER2*-amplified group, no significant prognostic IHC (ER, PR, HER2, and Ki67 global expression levels) indicators were found by the univariate analyses; therefore, only a set of ITH and FISH indicators along with the pathological variables were used in model 4. Models 5 and 6 were built with the same sets of variables as in the *HER2* non-amplified group. In model 4, higher values of HER2 MC contrast and CEP17 copy number indicators predicted better OS, while pN was associated with worse OS. The prognostic power of model 5 was increased by adding immune response indicators (likelihood ratio 29.03 of model 5 compared with 17.64 of model 4), where higher CD8 density in the tumor aspect of IZ predicted better OS. The latter indicator was also an independent factor of better OS in the context of worse OS predicted by pN status in model 6.

**Figure 1:**
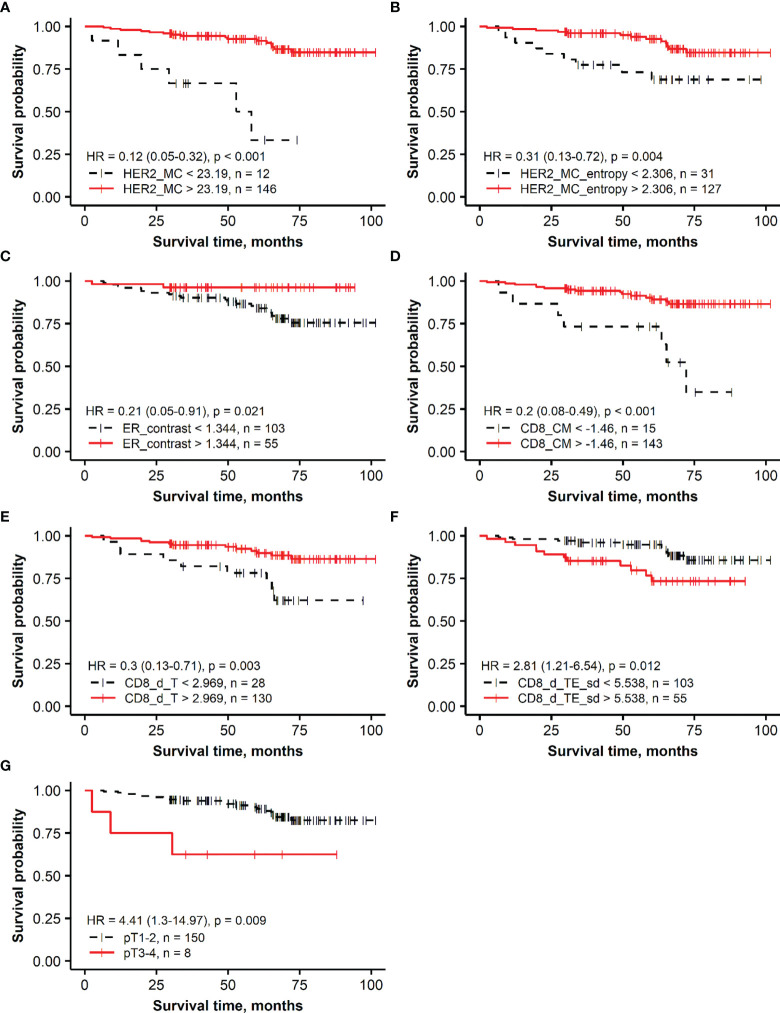
Kaplan–Meier survival plots representing the association of overall survival in the group of patients with *HER2* non-amplified breast cancer with independent prognostic indicators identified by multiple Cox regression analysis: **(A)** membrane completeness (HER2 MC), **(B)** membrane completeness entropy (HER2 MC entropy), **(C)** ER contrast, **(D)** center of mass for CD8 density (CD8_CM), **(E)** mean CD8 density in the tumor aspect (CD8_d_T), **(F)** SD of CD8 density in the tumor edge aspect (CD8_d_TE_sd), and **(G)** tumor stage (T).

**Figure 2:**
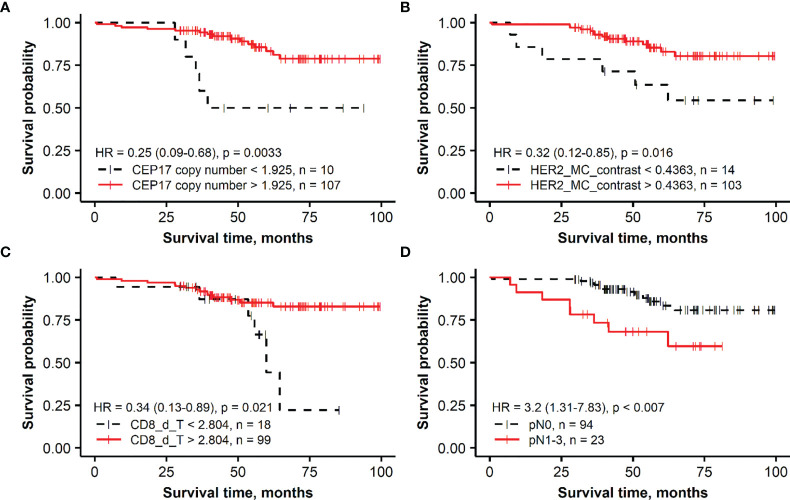
Kaplan–Meier survival plots representing the association of overall survival in the group of patients with *HER2*-amplified breast cancer with independent prognostic indicators identified by multiple Cox regression analysis: **(A)** CEP17 copy number, **(B)** membrane completeness contrast (HER2 MC contrast), **(C)** mean CD8 density in the tumor aspect (CD8_d_T), and **(D)** lymph node status (pN).

The Kaplan–Meier survival probability plots demonstrating an association between the prognostic factors and OS are presented for the *HER2* non-amplified and *HER2*-amplified groups in **Figures 1**, **2**, respectively.

### Combined CD8 Immunogradient Prognostic Score in *HER2* Non-Amplified Patient Group

**Figure 3:**
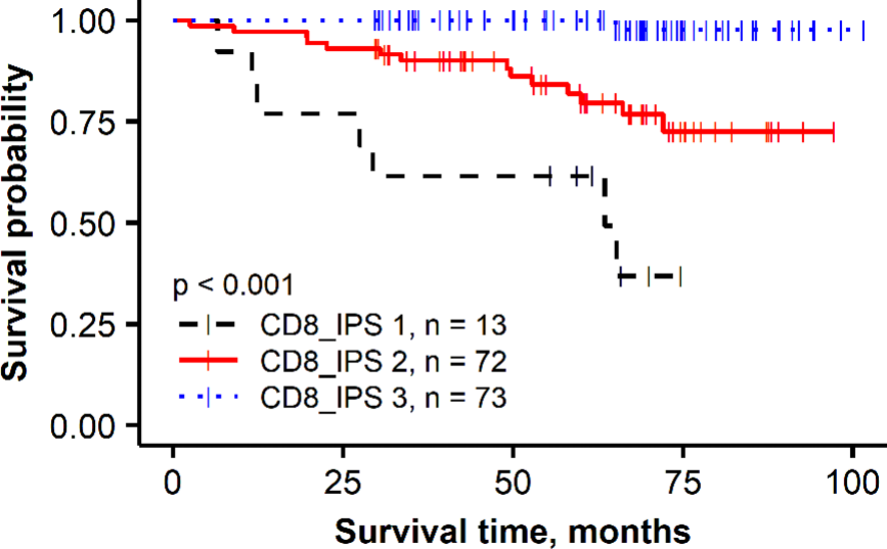
Kaplan–Meier plot for the overall survival of prognostic groups obtained by combined CD8 Immunogradient prognostic score (CD8_IPS) in HER2 non-amplified group.

To further assess the added prognostic value of the independent immune response features revealed by the multivariate regression analysis in the *HER2* non-amplified group, a combined CD8 Immunogradient prognostic score was calculated by summing corresponding scores (0/1) for each factor (CD8_CM, CD8_d_T, and CD8_d_TE_sd), assigning the score 1 for good or 0 for poor prognosis. The combined CD8 Immunogradient prognostic score allowed stratification of patients into three prognostic groups with 5-year OS probability of 98%, 80%, and 49% for the score of 3, 2, and 1, respectively (**Figure 3**). Of note, there were no patients with all three indicators assigned a score of 0.

## Discussion

In this study, we present prognostic models for patients with HER2 IHC borderline (2+) BC patients, based on the expression levels of ER, PR, Ki67, HER2, and CD8 densities in the tumor tissue assessed by DIA. These biomarkers were augmented by a set of computational indicators that quantify spatial aspects of ITH and tumor microenvironment. Importantly, the CD8 (immune response) indicators markedly strengthened the models in both the *HER2* non-amplified and *HER2*-amplified groups. Furthermore, these latter indicators outperformed pathological variables and enabled independent prognostic stratification of the *HER2* non-amplified BC patients.

DIA enables extraction of IHC data and spatial aspects from WSI with high capacity, not available by conventional IHC scoring. Additional processing of the DIA-generated data by hexagonal tiling enabled extraction of Haralick’s texture measures of ITH of the biomarkers of the prognostic value, as reported previously. In this study, we found that, of the IHC variables explored in the *HER2* non-amplified group, only HER2 expression percentage and HER2 MC were significantly associated with the patient outcome (*HR* = 0.25, *p* = 0.001 and *HR* = 0.12, *p* < 0.0001, respectively). Of note, HER2 MC was a stronger indicator than the proportion of HER2-positive tumor cells (as assessed by the HALO DIA) and served as an independent prognostic factor of better OS. Remarkably, two ITH indicators—HER2 MC entropy and ER contrast—showed an independent prognostic significance in the context of tumor stage status (**Table 3**, models 1 and 2). Similar findings of beneficial prognostic impact of higher HER2 MC were reported recently in early HR-positive BC patients, where better prognosis of higher HER2 expression was found in a univariate analysis (58). In another study, a trend of more favorable prognosis with respect to relapse-free survival has been shown for the ER-positive, *HER2* non-amplified tumors with higher levels of HER2 RNA (91).

HER2 MC status shows the status of HER2 expression, as HER2 protein is localized on the cell membrane. This means that HER2 MC entropy, which is indicative of MC spatial heterogeneity, also reveals information about the ITH of the HER2 protein expression. We observed a non-linear relationship between the HER2 MC and its spatial heterogeneity in our study (**Supplementary Figure 4**), represented by high ITH values in the middle range of the MC and lower ITH in the low and high ends of the MC variance. Similar dependencies between PR and Ki67 and their ITH indicators were previously reported (56–58); importantly, these studies demonstrated that the ITH indicators of Ki67 and PR expression enabled higher prognostic power than the expression rates *per se*. Our study extends this evidence by showing a greater prognostic value of ER ITH indicator than by the rate of its expression. Interestingly, ER contrast was the only ITH indicator of HR that provided the prognostic value in a univariate analysis (**Table 2**, *HR* = 0.21, *p* = 0.021) and in the multiple Cox regression models (**Table 3**, *HR* = 0.21, *p* = 0.0449, model 1, *HR* = 0.16, *p* = 0.0258, model 2, respectively). Haralick’s contrast (88) measures the spatial distribution of tumor cell subpopulations with different properties in the image. The associations of ER contrast with HER2 MC entropy and their inverse relation to ER expression were revealed by factor analysis (Factor 5, **Supplementary Figure 2** and **Supplementary Table 2**). This HER2&ER heterogeneity factor reflects the higher ITH of both HER2 and ER proteins in the tumors with decreased ER expression.

A majority of the patients with *HER2*-amplified tumors received adjuvant trastuzumab treatment (87, 74.4%). The OS of these patients is likely to have been impacted by the targeted therapy; therefore, the prognostic models obtained in this subgroup should be taken with caution. One can speculate that any potential effect of the targeted therapy could be related to our finding of ITH of HER2 expression, represented by the HER2 MC contrast indicator as independent predictor of better OS (**Table 3**, *HR* = 0.35, *p* = 0.0367, Model 4 and *HR* = 0.243, *p* = 0.0077, Model 5) but not by the HER2 MC indicator. The effect on better OS caused by a higher CEP17 copy number in this group is not clear, and it may be related to various treatment modalities applied in *HER2*-amplified BC patients. Several studies have reported an association between CEP17 copy number gain and responsiveness to anthracycline-based chemotherapy (92–94). Also, in addition to *HER2*, chromosome 17 includes other genes involved in BC pathogenesis and DNA repair, such as *BRCA1*, *TOP2A*, *TP53*, and *RAD51C* (95, 96); therefore, various abnormalities of chromosome 17 may affect prognosis and treatment response.

In this study, we tested the prognostic value of CD8 cell densities quantified by DIA in the tumor and stroma compartments and applied a recently proposed method, based on hexagonal grid analytics of the DIA data to compute CD8 local density profiles (Immunogradient) across automatically detected tumor–stroma IZ (86). This method actually tests if the immune cells reveal increasing densities towards the tumor at the tumor/host interface and therefore is expected to be more sensitive to capture “spatial behavior” of TILs. Indeed, higher CD8 cell densities in the tumor compartment were associated with better OS in univariate analyses in both patient subgroups (**Table 2**, *HR* = 0.37, *p* = 0.017 and *HR* = 0.38, *p* = 0.024 in the *HER2* non-amplified and *HER2*-amplified groups, respectively); however, they did not provide the independent prognostic value in our models. In contrast, three Immunogradient indicators provided the independent prognostic value in the non-amplified tumors: CD8 density in the tumor aspect of IZ (CD8_d_T) and positive IZ CD8 density gradient towards the tumor (CD8_CM) were associated with better OS, while the variance (SD) of CD8 density (CD8_d_TE_sd) along the TE predicted worse OS. A strong prognostic stratification was achieved by aggregating these three independent spatial properties of the CD8 cell distribution in the tumor microenvironment into a combined CD8 Immunogradient prognostic score; this represents an instance of computational augmentation of a single IHC biomarker (**Figure 3**). Remarkably, these three indicators were sufficient to predict OS independently of any other variables (**Table 3**, model 3) with statistical power obtained from pathology and IHC data supplemented with ITH indicators (**Table 3**, model 1). Finally, the prognostic power was doubled by adding the immune response indicators to the model (**Table 3**, model 2). Our findings are similar to the results presented in the study of Rasmusson et al. (86), where both CD8 density in the tumor aspect of IZ and CM for CD8 cell density within the IZ indicators were independent predictors of better OS in early HR-positive BC. Although several studies have reported a higher density of CD8 cells to be associated with a favorable prognosis in node-negative BC (97), or in combination with CD163 (98), other studies have shown an adverse prognostic effect of increased CD8 lymphocytes in patients with HR-positive/HER2-negative tumors (77, 99, 100) or reported no significant association between CD8 cells and patient outcome (79). These contradictory results in HR-positive BC may be related to different methodologies applied, lacking precision in the assessment of spatial aspects of TIL distributions within the tissues (100, 101). Recently, Dieci et al. (102) highlighted the need of deeper insight into the mechanisms on which the interaction between HR-positive/HER2-negative BC tumor and immune cells relies, as various factors such as menopausal status, estrogen levels, and endocrine treatments may be involved in the modulation of the tumor microenvironment (102). Therefore, methods with appropriate discriminatory spatial precision are needed to expose the prognostic role of TILs in luminal-like BC.

We did not find significant differences in CD8 cell densities between the *HER2*-amplified and non-amplified groups, which could be explained by the fact that the *HER2*-amplified group was composed of both molecular subgroups showing HER2 positivity, namely, luminal B and HER2-enriched. Previous studies reported that HER2-enriched subtype is more immunogenic than the luminal B (103). In our study, the only immune response indicator—density of CD8 in the T aspect of IZ—provided an independent association with better OS in HER2-amplified BC patients (**Table 3**, models 5 and 6). Extensive TIL infiltration has been associated with better outcomes (pathological complete response, event-free survival, and disease-free survival) in HER2-positive BC (70, 104, 105). However, studies evaluating the prognostic significance of CD8 TILs reported conflicting results (77, 79, 100, 106, 107), suggesting that the association between CD8 cells and prognosis depends on lymphocyte types, their tissue location, analysis methods, and other factors. Indeed, the interaction between immune system and tumor as well as prognostic effects of TILs in HER2-positive BC is impacted by various combined therapy modalities, including anti-HER2 therapy, chemotherapy, and hormonal therapy (108). Trastuzumab therapy effect depends on immune response (109), it has both cytotoxic and immunological effects on tumor cells (110–112), and better therapeutic efficacy is achieved in tumors with high TILs (113–115). However, this was not confirmed by other studies (116, 117).

Our study has limitations, related to its retrospective and monocentric design and lack of well-structured information about applied therapies and responses achieved. In particular, it is relevant to the prognostic modeling in the HER2-positive patient cohort.

In conclusion, we present prognostic OS models based on computational ITH, tumor microenvironment, and immune response indicators of the IHC biomarkers in HER2 IHC 2+ borderline BC patients. The ITH indicators (HER2 MC entropy and ER contrast in FISH-negative and HER2 MC contrast in FISH-positive tumors) provided an independent contribution to predict better OS. In FISH-negative tumors, antitumor immune response, assessed by the CD8 IZ Immunogradient indicators, provided prognostic stratification independent and superior to other pathology and IHC variables.

## Data Availability Statement

The raw data supporting the conclusions of this article will be made available by the authors, without undue reservation.

## Ethics Statement

The studies involving human participants were reviewed and approved by Lithuanian Bioethics Committee. The patients/participants provided their written informed consent to participate in this study.

## Author Contributions

GR, ArL, and AR designed the study. GR in collaboration with AiL collected the samples and participated in IHC slide staining. GR, RG, and RA performed digital image analyses. GR and RA performed statistical analyses. DZ participated in the statistical analysis of CD8. GR in collaboration with AR and ArL drafted essential parts of the manuscript. All authors reviewed the analysis results and read and approved the final manuscript.

## Funding

This project had received funding from the European Social Fund, project No. 09.3.3-LMT-K-712-01-0139, under grant agreement with the Research Council of Lithuania.

## Conflict of Interest

The authors declare that the research was conducted in the absence of any commercial or financial relationships that could be construed as a potential conflict of interest.

## Publisher’s Note

All claims expressed in this article are solely those of the authors and do not necessarily represent those of their affiliated organizations, or those of the publisher, the editors and the reviewers. Any product that may be evaluated in this article, or claim that may be made by its manufacturer, is not guaranteed or endorsed by the publisher.
