# Supplementary material for: Intratumoral Heterogeneity and Immune Response Indicators to Predict Overall Survival in a Retrospective Study of HER2-Borderline (IHC 2+) Breast Cancer Patients
Source: Front Oncol. 2021 Nov 11;11:774088. doi: 10.3389/fonc.2021.774088 (PMC8631965; doi:10.3389/fonc.2021.774088)
Supplement: Supplementary file 1 [file DataSheet_1.zip › Supplementary Figure 1.docx]

Supplementary Material


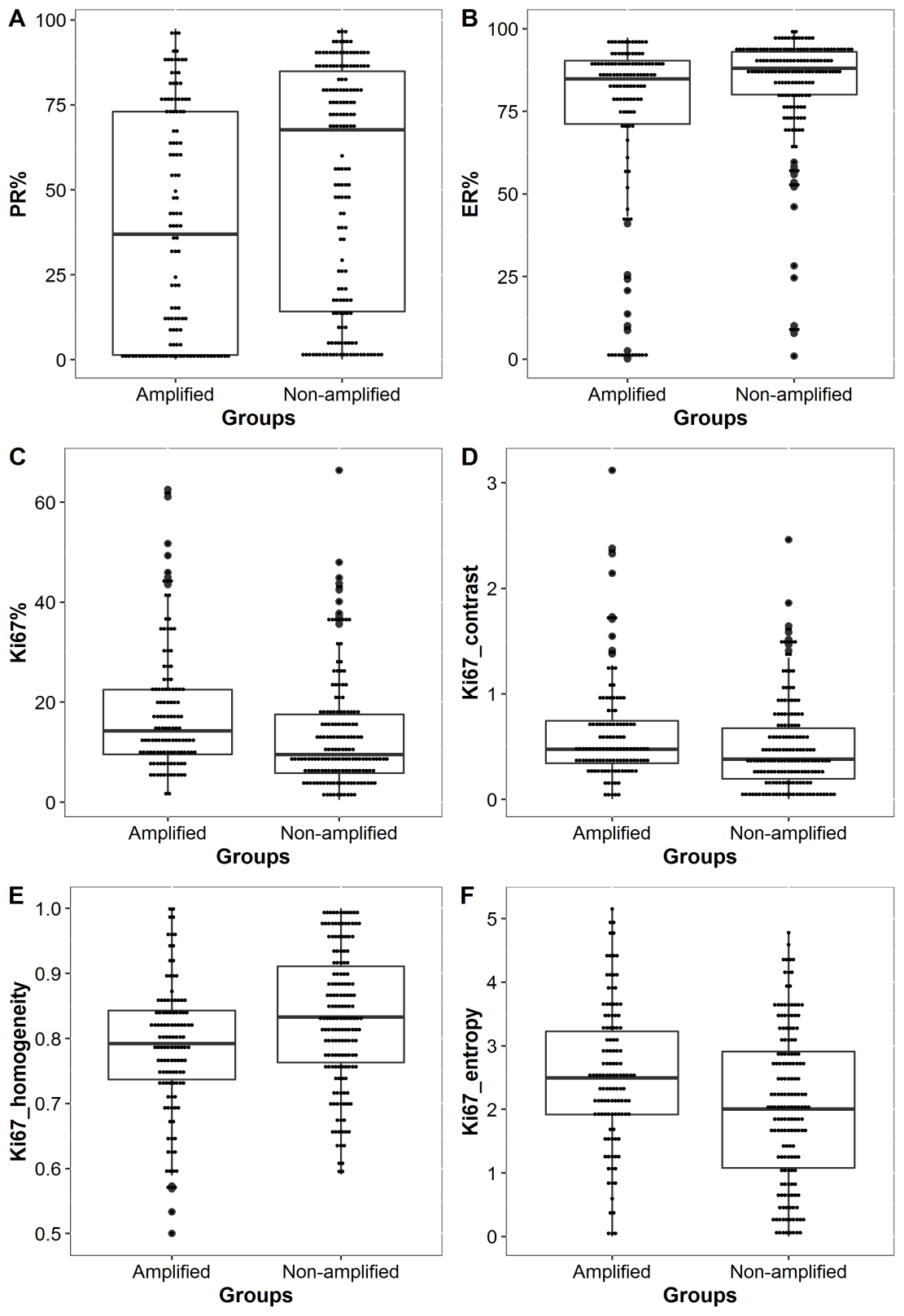


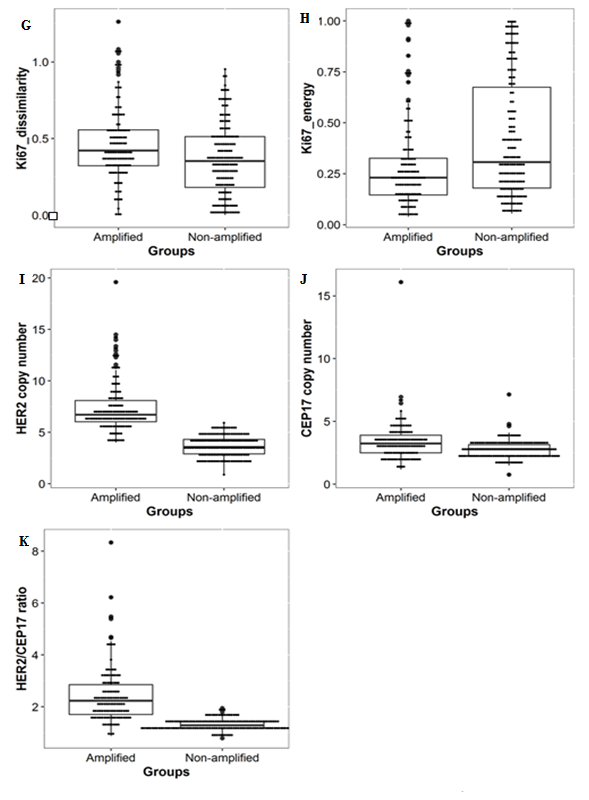


**Supplementary Figure 1:** **The variance plots of the significant differences between variables in *HER2* non-amplified and amplified groups: (A)** PR%, **(B)** ER%, **(C)** Ki67%, **(D)** Ki67 contrast, **(E)** Ki67 homogeneity, **(F)** Ki67 entropy, **(G)** Ki67 dissimiliarity, **(H)** Ki67 energy **(I)** *HER2* copy number, **(J)** CEP17 copy number, **(K)** *HER2*/CEP17 ratio
