# Supplementary material for: Intratumoral Heterogeneity and Immune Response Indicators to Predict Overall Survival in a Retrospective Study of HER2-Borderline (IHC 2+) Breast Cancer Patients
Source: Front Oncol. 2021 Nov 11;11:774088. doi: 10.3389/fonc.2021.774088 (PMC8631965; doi:10.3389/fonc.2021.774088)
Supplement: Supplementary file 1 [file DataSheet_1.zip › Supplementary Figure 3.docx]

Supplementary Material


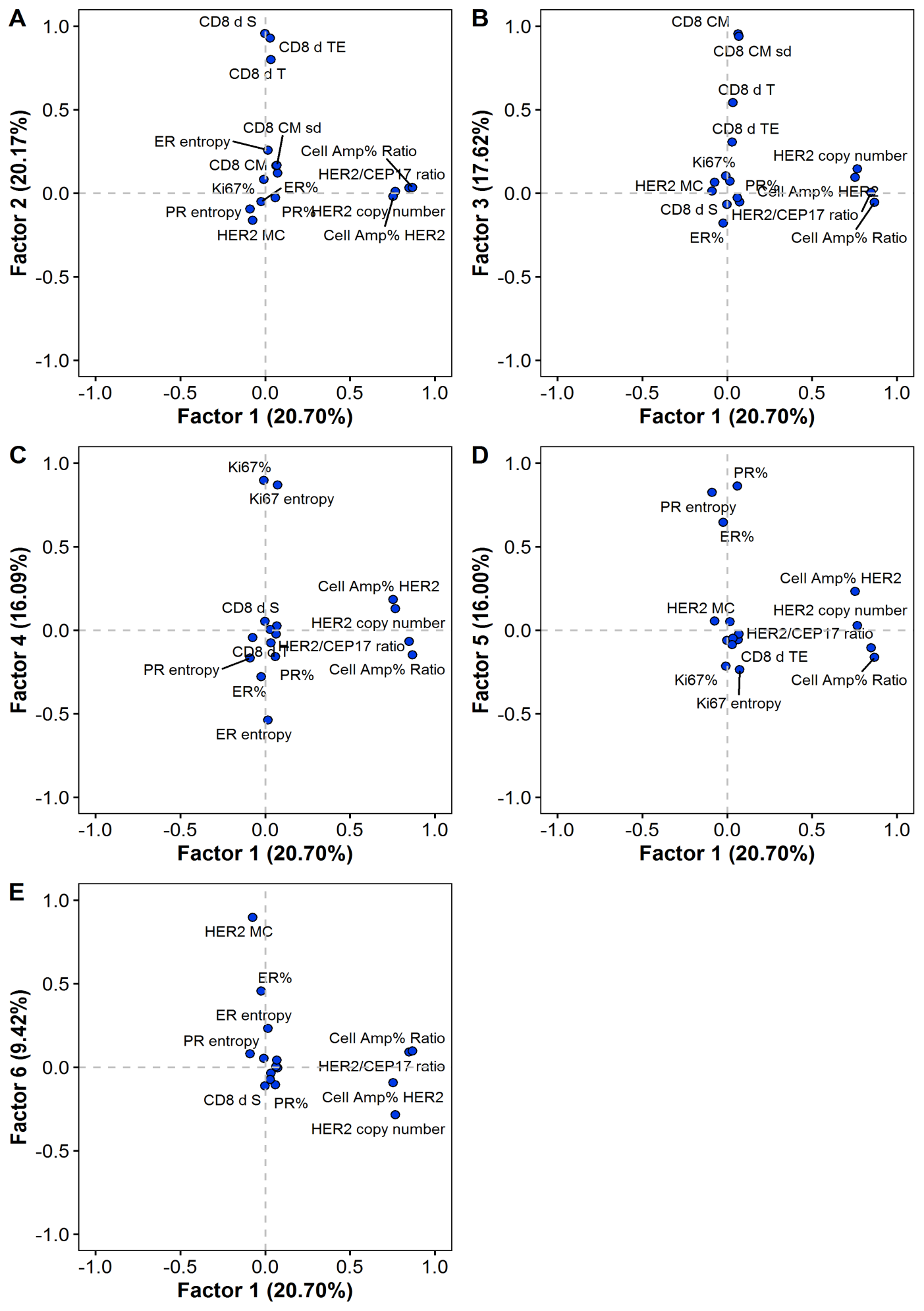


**Supplementary Figure 3: Rotated factor pattern in *HER2*-amplified patients group: (A) the loadings of factors 1 and 2, (B) factors 1 and 3, (C) factors 1 and 4, (D) factors 1 and 5 and (E) factors 1 and 6 are plotted:** Cell_Amp_%_Ratio – percentage of amplified cells calculated from *HER2*/CEP17 ratio, Cell_Amp_%_*HER2* – percentage of amplified cells calculated by HER2 signals only, CM – center of mass, CM_sd – standard deviation for center of mass, d_S – density in the stroma aspect of IZ, d_TE – density in the tumor edge aspect of IZ, d_T – density in the tumor aspect of IZ, MC – membrane completeness.
