# Supplementary material for: Intratumoral Heterogeneity and Immune Response Indicators to Predict Overall Survival in a Retrospective Study of HER2-Borderline (IHC 2+) Breast Cancer Patients
Source: Front Oncol. 2021 Nov 11;11:774088. doi: 10.3389/fonc.2021.774088 (PMC8631965; doi:10.3389/fonc.2021.774088)
Supplement: Supplementary file 1 [file DataSheet_1.zip › Supplementary Figure 4.docx]

Supplementary Material


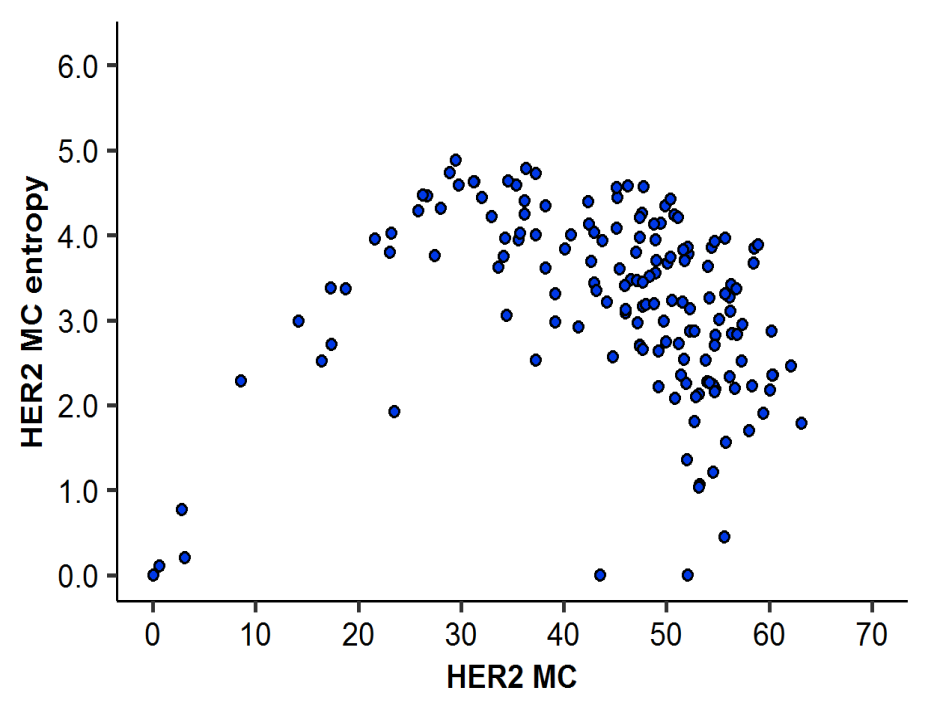


Supplementary Figure 4: Correlation of HER2 membrane completeness (MC) and its entropy. The scatterplot displays non-linear relationship between two variables that was positive at lower-left-to-upper-right part (less uniform membrane staining) and negative at upper-left-to-lower-right part (more uniform membrane staining).
