# Supplementary material for: Intratumoral Heterogeneity and Immune Response Indicators to Predict Overall Survival in a Retrospective Study of HER2-Borderline (IHC 2+) Breast Cancer Patients
Source: Front Oncol. 2021 Nov 11;11:774088. doi: 10.3389/fonc.2021.774088 (PMC8631965; doi:10.3389/fonc.2021.774088)
Supplement: Supplementary file 1 [file DataSheet_1.zip › Supplementary Table 1.DOCX]

Supplementary Material

|  | **Non-amplified *HER2* status** | | | | | **Amplified *HER2* status** | | |  |
| --- | --- | --- | --- | --- | --- | --- | --- | --- | --- |
|  | **Mean** | | **SD** | **Median** | | **Mean** | **SD** | **Median** | ***p*-value*** |
| **IHC indicators** |  | |  | |  |  |  |  |  |
| HER2% | | 29.37 | 13.96 | | 28.07 | 31.76 | 15.76 | 29.29 | 0.414 |
| HER2_MC | 44.55 | | 13.02 | | 48.16 | 44.46 | 11.85 | 48.26 | 0.6574 |
| ER% | 82.72 | | 17.43 | | 87.99 | 71.39 | 30.35 | 84.74 | 0.0017* |
| PR% | 51.76 | | 34.79 | | 67.6 | 38.65 | 34.12 | 36.91 | 0.0007* |
| Ki67% | 13.54 | | 11.26 | | 9.49 | 18.19 | 12.52 | 14.24 | <0.0001* |
| **FISH indicators** |  | |  | |  |  |  |  |  |
| *HER2* copy number | 3.66 | | 1.03 | | 3.55 | 7.37 | 2.48 | 6.7 | <0.0001* |
| CEP17 copy number | 2.78 | | 0.72 | | 2.78 | 3.39 | 1.62 | 3.23 | <0.0001* |
| *HER2*/CEP17 ratio | 1.32 | | 0.21 | | 1.28 | 2.43 | 1.12 | 2.23 | <0.0001* |
| **Immune response indicators** |  | |  | |  |  |  |  |  |
| CD8_S | 135.06 | | 132.62 | | 94.87 | 154.44 | 158.38 | 96.65 | 0.2457 |
| CD8_T | 109.09 | | 127.62 | | 60.9 | 130.89 | 176.74 | 72.25 | 0.6105 |
| CD8_CM | ˗0.61 | | 0.67 | | ˗ 0.64 | ˗0.56 | 0.63 | ˗0.56 | 0.5245 |
| CD8_CM_sd | ˗0.64 | | 0.48 | | ˗ 0.60 | ˗0.56 | 0.45 | ˗ 0.53 | 0.1917 |
| CD8_ID | 3.48 | | 4.56 | | 2.14 | 3.42 | 3.48 | 2.19 | 0.5683 |
| CD8_d_S | 173.05 | | 160.9 | | 122.34 | 193.36 | 178.33 | 131.83 | 0.2396 |
| CD8_d_S_sd | 268.05 | | 157.59 | | 228.82 | 286.7 | 58.5 | 245.14 | 0.2577 |
| CD8_d_TE | 158.41 | | 173.98 | | 101.14 | 184.53 | 201.45 | 109.35 | 0.1746 |
| CD8_d_TE_sd | 229.46 | | 145.48 | | 189.56 | 256.32 | 142.92 | 226.86 | 0.0524 |
| CD8_d_T | 100.92 | | 125.2 | | 52.72 | 121.97 | 181.21 | 57.93 | 0.618 |
| CD8_d_T_sd | 153.68 | | 115.77 | | 122.54 | 173.27 | 125.91 | 141.08 | 0.1592 |
| **Intratumoral heterogeneity indicators** |  | |  | |  |  |  |  |  |
| HER2_contrast | 1.25 | | 0.8 | | 1.07 | 1.32 | 0.77 | 1.18 | 0.3803 |
| HER2_dissimilarity | 0.71 | | 0.27 | | 0.69 | 0.73 | 0.26 | 0.74 | 0.4768 |
| HER2_entropy | 3.71 | | 1.12 | | 3.9 | 3.84 | 0.94 | 3.99 | 0.4299 |
| HER2_energy | 0.16 | | 0.19 | | 0.1 | 0.14 | 0.14 | 0.09 | 0.5662 |
| HER2_homogeneity | 0.69 | | 0.1 | | 0.69 | 0.69 | 0.09 | 0.67 | 0.5477 |
| HER2_AshD | 4.21 | | 4.6 | | 2.37 | 3.7 | 3.52 | 2.32 | 0.8704 |
| HER2_MC_contrast | 0.89 | | 0.5 | | 0.82 | 0.96 | 0.51 | 0.86 | 0.4157 |
| HER2_MC_dissimilarity | 0.57 | | 0.23 | | 0.58 | 0.6 | 0.22 | 0.58 | 0.5569 |
| HER2_MC_entropy | 3.18 | | 1.07 | | 3.36 | 3.35 | 0.9 | 3.47 | 0.3976 |
| HER2_MC_energy | 0.22 | | 0.2 | | 0.15 | 0.19 | 0.13 | 0.15 | 0.602 |
| HER2_MC_homogeneity | 0.74 | | 0.09 | | 0.74 | 0.73 | 0.08 | 0.73 | 0.642 |
| HER2_MC_AshD | 2.07 | | 1.18 | | 1.84 | 2.1 | 1.17 | 1.77 | 0.9688 |
| ER_contrast | 1.33 | | 1.36 | | 0.91 | 1.25 | 1.13 | 0.96 | 0.9067 |
| ER_dissimilarity | 0.58 | | 0.39 | | 0.54 | 0.57 | 0.39 | 0.53 | 0.9359 |
| ER_entropy | 2.7 | | 1.51 | | 2.68 | 2.7 | 1.57 | 2.81 | 0.8607 |
| ER_energy | 0.35 | | 0.28 | | 0.25 | 0.36 | 0.31 | 0.23 | 0.8296 |
| ER_homogeneity | 0.77 | | 0.13 | | 0.77 | 0.77 | 0.14 | 0.76 | 0.8022 |
| ER_AshD | 4.67 | | 7.15 | | 1.92 | 3.63 | 4.52 | 1.87 | 0.5756 |
| PR_contrast | 1.66 | | 1.38 | | 1.52 | 1.55 | 1.52 | 1.17 | 0.229 |
| PR_dissimilarity | 0.66 | | 0.45 | | 0.67 | 0.62 | 0.52 | 0.58 | 0.2751 |
| PR_entropy | 3.06 | | 1.8 | | 3.41 | 2.82 | 2.08 | 3.13 | 0.4117 |
| PR_energy | 0.35 | | 0.36 | | 0.19 | 0.42 | 0.39 | 0.25 | 0.4303 |
| PR_homogeneity | 0.75 | | 0.16 | | 0.73 | 0.77 | 0.18 | 0.77 | 0.3256 |
| PR_AshD | 2.3 | | 1.87 | | 1.84 | 2.3 | 2.12 | 1.82 | 0.558 |
| Ki67_contrast | 0.5 | | 0.44 | | 0.38 | 0.63 | 0.51 | 0.47 | 0.0034* |
| Ki67_dissimilarity | 0.36 | | 0.24 | | 0.35 | 0.47 | 0.24 | 0.42 | 0.001* |
| Ki67_entropy | 2.05 | | 1.22 | | 2 | 2.55 | 1.11 | 2.49 | 0.0013* |
| Ki67_energy | 0.42 | | 0.3 | | 0.31 | 0.29 | 0.22 | 0.23 | 0.0006* |
| Ki67_homogeneity | 0.83 | | 0.11 | | 0.83 | 0.78 | 0.1 | 0.79 | 0.0004* |
| Ki67_AshD | 4.36 | | 7.25 | | 1.87 | 5.06 | 7.44 | 2.08 | 0.9274 |
|  |  | |  | |  | |  |  |  |

**Supplementary Table 1:** **Summary statistics of IHC and FISH indicators in *HER2* non-amplified and amplified tumors:** AshD – Ashman’s D, CEP17 – centromere enumeration probe for chromosome 17, CM – center of mass, CM_sd – standard deviation for center of mass, d – density, ID – immunodrop, d_S – density in the stroma aspect of IZ, d_S_sd – standard deviation in the stroma aspect of IZ, d_T – density in the tumor aspect of IZ, d_T_sd – standard deviation in the tumor aspect of IZ, d_TE – density in the tumor edge aspect of IZ, d_TE_sd – standard deviation in the tumor edge aspect of IZ, MC – membrane completeness, sd – standard deviation, S – stroma area, T – tumor area. * *p*-value < 0.05 is considered significant.
