# Supplementary material for: Intratumoral Heterogeneity and Immune Response Indicators to Predict Overall Survival in a Retrospective Study of HER2-Borderline (IHC 2+) Breast Cancer Patients
Source: Front Oncol. 2021 Nov 11;11:774088. doi: 10.3389/fonc.2021.774088 (PMC8631965; doi:10.3389/fonc.2021.774088)
Supplement: Supplementary file 1 [file DataSheet_1.zip › Supplementary Table 2.DOCX]

Supplementary Material

|  | **Factor1** | **Factor2** | **Factor3** | **Factor4** | **Factor5** | **Factor6** |
| --- | --- | --- | --- | --- | --- | --- |
| **HER2_MC** | 0.004 | -0.00146 | 0.07852 | 0.35257 | -0.26555 | -0.28593 |
| **ER%** | -0.20888 | -0.13154 | -0.11013 | -0.07471 | -0.74078 | 0.17116 |
| **PR%** | 0.02041 | 0.02155 | 0.07716 | -0.11113 | -0.14067 | 0.83128 |
| **Ki67%** | 0.06169 | 0.02566 | 0.01537 | 0.93836 | 0.04091 | -0.02409 |
| ***HER2* copy number** | -0.00106 | 0.85792 | 0.01178 | 0.09921 | -0.00514 | 0.08871 |
| ***HER2*/CEP17 ratio** | -0.09212 | 0.76224 | -0.08529 | -0.00583 | 0.11843 | -0.19984 |
| **Cell_Amp_%_Ratio** | 0.01405 | 0.81957 | -0.03021 | -0.02996 | 0.02641 | -0.05541 |
| **Cell_Amp_%_*HER2*** | 0.09298 | 0.77203 | 0.08414 | 0.07743 | -0.0922 | 0.18527 |
| **CD8_CM** | 0.11846 | -0.00008 | 0.95758 | -0.06375 | 0.11479 | 0.00595 |
| **CD8_CM_sd** | 0.15018 | -0.0257 | 0.94619 | 0.02384 | 0.10731 | -0.00779 |
| **CD8_d_S** | 0.95826 | 0.00004 | -0.06559 | 0.07111 | 0.04891 | 0.01987 |
| **CD8_d_TE_sd** | 0.9416 | 0.01979 | 0.19478 | 0.08053 | 0.11192 | 0.0268 |
| **CD8_d_T** | 0.78801 | 0.02647 | 0.52016 | 0.00764 | 0.16054 | 0.07223 |
| **HER2_MC_entropy** | -0.12141 | 0.04169 | 0.07611 | 0.04217 | 0.637 | 0.19092 |
| **ER_contrast** | 0.35043 | -0.16746 | 0.10572 | -0.03961 | 0.63577 | 0.02773 |
| **PR_entropy** | 0.05795 | -0.00499 | -0.05067 | 0.08308 | 0.20263 | 0.77971 |
| **Ki67_entropy** | 0.07246 | 0.09913 | -0.09604 | 0.92592 | 0.11394 | 0.0516 |

Supplementary Table 2: Rotated factor pattern of IHC, FISH, immune response and intratumoral heterogeneity indicators of *HER2* non-amplified breast cancer cohort: Cell_Amp_%_Ratio – percentage of amplified cells calculated from *HER2*/CEP17 ratio, Cell_Amp_%_*HER2* – percentage of amplified cells calculated by *HER2* signal only, CM – center of mass, CM_sd – standard deviation for center of mass, d_S – density in the stroma aspect of IZ, d_TE_sd – standard deviation in the tumor edge aspect of IZ, d_T – density in the tumor aspect of IZ, MC – membrane completeness.
