# Supplementary material for: Intratumoral Heterogeneity and Immune Response Indicators to Predict Overall Survival in a Retrospective Study of HER2-Borderline (IHC 2+) Breast Cancer Patients
Source: Front Oncol. 2021 Nov 11;11:774088. doi: 10.3389/fonc.2021.774088 (PMC8631965; doi:10.3389/fonc.2021.774088)
Supplement: Supplementary file 1 [file DataSheet_1.zip › Supplementary Table 3.DOCX]

Supplementary Material

|  | **Factor1** | **Factor2** | **Factor3** | **Factor4** | **Factor5** | **Factor6** |
| --- | --- | --- | --- | --- | --- | --- |
| **HER2_MC** | -0.07292 | -0.16329 | 0.06454 | -0.04482 | 0.05408 | 0.89483 |
| **ER%** | -0.02294 | -0.0512 | -0.17988 | -0.27879 | 0.64454 | 0.45539 |
| **PR%** | 0.06011 | -0.02826 | -0.02778 | -0.15932 | 0.86288 | -0.10557 |
| **Ki67%** | -0.00678 | 0.08184 | 0.10232 | 0.89646 | -0.21699 | 0.05264 |
| ***HER2* copy number** | 0.76691 | 0.00983 | 0.14446 | 0.12767 | 0.02636 | -0.28484 |
| ***HER2*/CEP17 ratio** | 0.84687 | 0.031 | 0.00234 | -0.06786 | -0.10615 | 0.09039 |
| **Cell_Amp_%_Ratio** | 0.86803 | 0.032 | -0.05485 | -0.14832 | -0.16273 | 0.0967 |
| **Cell_Amp_%_*HER2*** | 0.75402 | -0.02063 | 0.09449 | 0.18281 | 0.23158 | -0.09335 |
| **CD8_CM** | 0.06377 | 0.16365 | 0.95287 | -0.02466 | -0.05824 | 0.00235 |
| **CD8_CM_sd** | 0.06845 | 0.16664 | 0.93684 | 0.02486 | -0.02446 | 0.04156 |
| **CD8_d_S** | -0.00162 | 0.9541 | -0.06773 | 0.05268 | -0.06251 | -0.11291 |
| **CD8_d_TE** | 0.03009 | 0.92767 | 0.30549 | 0.0042 | -0.08842 | -0.0748 |
| **CD8_d_T** | 0.03424 | 0.79922 | 0.54139 | -0.07667 | -0.04875 | -0.0358 |
| **ER_entropy** | 0.01542 | 0.25721 | 0.0705 | -0.53803 | 0.04917 | 0.23142 |
| **PR_entropy** | -0.08912 | -0.09608 | 0.01222 | -0.1678 | 0.82355 | 0.08013 |
| **Ki67_entropy** | 0.07367 | 0.12003 | -0.05431 | 0.86915 | -0.23736 | -0.0051 |

Supplementary Table 3: Rotated factor pattern of IHC, FISH, immune response and intratumoral heterogeneity indicators of *HER2*-amplified breast cancer cohort: Cell_Amp_%_Ratio – percentage of amplified cells calculated from *HER2*/CEP17 ratio, Cell_Amp_%_HER2 – percentage of amplified cells calculated by *HER2* signal only, CM – center of mass, CM_sd – standard deviation for center of mass, d_S – density in the stroma aspect of IZ, d_TE – density in the tumor edge aspect of IZ, d_T – density in the tumor aspect of IZ, MC – membrane completeness.
